# Dining comfort in elderly care facility dining rooms and influencing factors before and after the outbreak of the COVID-19 pandemic

**DOI:** 10.3389/fpsyg.2023.1106741

**Published:** 2023-03-03

**Authors:** Jingyi Mu, Jian Kang

**Affiliations:** ^1^Key Laboratory of Cold Region Urban and Rural Human Settlement Environment Science and Technology, School of Architecture, Ministry of Industry and Information Technology, Harbin Institute of Technology, Harbin, China; ^2^Institute for Environmental Design and Engineering, The Bartlett, University College London, London, United Kingdom

**Keywords:** COVID-19, elderly, care facilities, dining room, dining comfort, dining modes

## Abstract

**Introduction:**

The COVID-19 pandemic has changed dining modes in elderly care facilities. This study explores the relationship between the dining environment of four elderly care facilities and the sensitivity of the elderly residents to it before and after the outbreak of the COVID-19 pandemic.

**Methods:**

The study examined differences in subjective comfort levels by measuring the facilities’ physical environment, analysing dining behavior, and surveying the elderly residents. And the study examined how the interaction between the physical environment factors, demographic factors, and dining mode affected the residents’ evaluation of their dining comfort.

**Results:**

(1) The physical environmental parameters of the four dining rooms differed between the pre- and post-epidemic periods, as shown by increased Sound Pressure Level (SPL), humidity, and temperature levels. (2) The residents’ evaluations of physical environment comfort also changed after the outbreak of the COVID-19 pandemic. The subjective comfort levels of the ‘dining with baffle’ and ‘dining across a seat’ modes decreased, though the level of the former was slightly higher than that of the latter. The elderly had stronger SPL tolerance in the dining with baffle mode and dining across a seat mode, and their subjective comfort levels for thermal environment and air quality were higher in the dining across a seat mode. (3) When dining time, crowd density, and communication frequency were kept equal, the subjective comfort level of the elderly in the dining with baffle mode and dining across a seat mode was lower than that in the ‘normal’ dining mode, when the level in the dining with baffle mode was lower than that in the dining across a seat mode. (4) Differences were observed in subjective comfort levels according to age, education level, and residence duration across the dining modes.

**Discussion:**

The need for changes in dining modes during the COVID-19 isolation period require dining rooms in elderly care facilities to design their physical environments in a way that improves dining comfort for the elderly.

## 1. Introduction

The COVID-19 pandemic has changed people’s living environments and heightened their risk perception (Cori et al., 2020; Xie et al., 2020). The most obvious change is the need to isolate and evacuate crowded places to stop the spread of the virus. Many shopping malls, dining rooms, and other crowded places have been closed, while people are staying behind closed doors and are working, studying, and living at home. The COVID-19 pandemic has brought great challenges to the world’s medical and nursing practitioners. In an era of accelerating aging, the elderly, as a group highly susceptible to infectious diseases, are of key concern regarding COVID-19. Therefore, elderly care facilities have taken necessary measures to reduce COVID-19 infection rates (Comas-Herrera et al., 2020; Iaboni et al., 2020). The COVID-19 pandemic has caused elderly care facilities to change the dining room modes of their residents. Common protection measures used in dining rooms inside public institutions include transparent baffles at tables or seating arranged across seats to avoid having people face each other. These measures have changed the dining environments of Chinese elderly care facilities, and these changes have a corresponding impact on the dining comfort of their elderly residents.

Elderly and young people differ significantly in terms of dining because of differences in age, physical fitness, and other factors. A study of ‘silver consumers’ in Malaysia found that elderly people living in households were more conscious of food quality and service when eating out (Bakar et al., 2020). Older people living in geriatric care homes will also expect a restaurant-style experience, but will value stable table companions over flexibility (Kenkmann and Hooper, 2012). People’s environmental perceptions have been affected by changes in living habits since the COVID-19 outbreak due to lockdown conditions, social distancing, reduced enclosed space capacity, and government-imposed curfews. Many students are required to take online classes at home, and differences in noise, light, temperature, and other factors in their environments affect their academic performance (Realyvásquez-Vargas et al., 2020). Due to the closure of their workplace, many employees are working in home offices, and the homes’ indoor environment is also having an impact on their environmental perceptions. The comfort of a person’s physical environment affects their productivity, and the visual factor is the most critical variable (Salamone et al., 2021). The elderly are highly susceptible to COVID-19 infection, and their eating behavior has changed more dramatically than that of any other groups due to the adoption of isolation measures (Damayanthi and Prabani, 2021). A qualitative survey of chronically ill elderly people in South Korea found that, after the start of the COVID-19 pandemic, older people began to cook at home or use delivery services instead of eating out, and the closure of restaurants and catering establishments increased the difficulty of accessing food for them (Kim et al., 2021). Eating alone, reduced physical activity, and anxiety/stress about possible COVID-19 infection can also lead to decreased appetite in older adults, which can adversely affect their dietary health (Visser et al., 2020). Amid the COVID-19 pandemic, the elderly are more sensitive to environmental perceptions than are other groups, and are more afraid to use public spaces, which imposes higher requirements for their design (Fabisiak et al., 2020). This is especially true for dining environments, which have significant impacts on the comfort of the elderly.

Diet is directly related to nutritional acquisition and is very important for the health of the elderly; the overall survival rate of the elderly is strongly related to the availability of a healthy diet (Trichopoulou et al., 1995). Healthy eating in older adults is influenced by underlying psychological and social factors that may moderate the effects of age (Bloom et al., 2017). For example, food intake and choice are affected by the environmental atmosphere (Stroebele and De Castro, 2004). Environmental factors may influence the food choices and eating behaviors of the growing population of community-dwelling elderly, and providing gathering places that provide high nutrition is important for social support and ensuring access to affordable healthy food (Sylvie et al., 2013). The nutrient intake of residents at elderly care facilities is both a clinical and quality-of-life issue, affecting physical and mental health and overall well-being (Morley and Silver, 1995; Pauly et al., 2007). Therefore, the dietary health of the elderly, including those in elderly care facilities, requires specific conditions. The elderly are highly susceptible to infectious diseases and account for the majority of patients with severe COVID-19 symptoms. The COVID-19 pandemic has caused many negative effects on the elderly, including unemployment and economic difficulties caused by the decline of savings, the shortage of various medical care services, and increased anxiety and depression (Morrow-Howell et al., 2020). A balanced diet is a very important part of maintaining good physical health for the elderly, and a comfortable dining environment is important for their emotional health (Ju et al., 2014). Dining comfort is affected by the dining room environment (Liu and Jang, 2009). Studies have found that adjusting noise and lighting levels at meals can increase food intake and improve nutrition in people with Alzheimer’s (McDaniel et al., 2001). The COVID-19 pandemic has caused dining rooms to change their dining modes, while changes in dining environments, communication and hygiene, and contactless functions of dining rooms have made customers feel comfortable eating in them (Jeong et al., 2022). Customers eating in regular dining rooms are more inclined to sit at a safe distance rather than in a partition (Wang et al., 2021). However, the elderly living in care facilities have few external dining room options, if any. Most eat in the dining rooms inside their care facilities, especially given the isolation requirements of the COVID-19 pandemic. Several measures have been taken in old-age care institutions to ensure the safety of their elderly residents and reduce their infection rates. The COVID-19 pandemic has caused elderly care facilities to change dining modes in their dining rooms. Using baffles and arranging for cross-seating are among the common measures employed to reduce cross-infection. These changes have corresponding impacts on the subjective comfort levels of the elderly.

Many public places, such as classrooms (Triyason et al., 2020), have adopted new designs to adapt to the normalization of the COVID-19 pandemic or have employed measures specific to the pandemic. Some of China’s COVID-19-related regulations are likely to remain in place, especially for vulnerable groups such as the elderly. In view of the importance of diet and dining for the health of the elderly, it is worth studying the impact of dining room isolation measures on the dining comfort of the elderly living in care facilities. Few studies have examined how dining modes in elderly care facilities affect the subjective comfort levels of elderly residents. Therefore, this study investigates four elderly care facilities in northeast China to explore ways to provide a more comfortable dining environment for the elderly while ensuring safety during the COVID-19 pandemic. To this end, this study focuses on four questions:

- What is the dining rooms environment (including acoustics, lighting, heat, air quality, and odor) like in elderly care facilities with different dining modes since the outbreak of the COVID-19 pandemic?
- What changes have occurred in how residents evaluate the physical environment comfort of dining rooms in elderly care facilities since the start of the COVID-19 pandemic outbreak?
- What impact do differences in dining mode have on the elderly in elderly care facilities before and after the start of the COVID-19 pandemic?
- What impact do demographic factors have on the dining comfort of the elderly with different eating patterns before and after the start of the COVID-19 pandemic?

## 2. Methods

This study measured the physical environments of dining rooms using different dining modes in elderly care facilities before and after the outbreak of COVID-19, focusing on Sound Pressure Level (SPL), intensity of illumination, brightness, temperature, humidity, air quality, and environment; the study also examined the dining behavior of the elderly eating in different dining modes, focusing on dining time, communication frequency, and population density. The study also conducted a questionnaire survey on factors related to the dining rooms’ physical environment for each dining mode. Finally, the study examined how the interaction between the physical environment factors, demographic factors, and dining mode affected the residents’ evaluation of their dining comfort.

### 2.1. Sites

**Figure 1:**
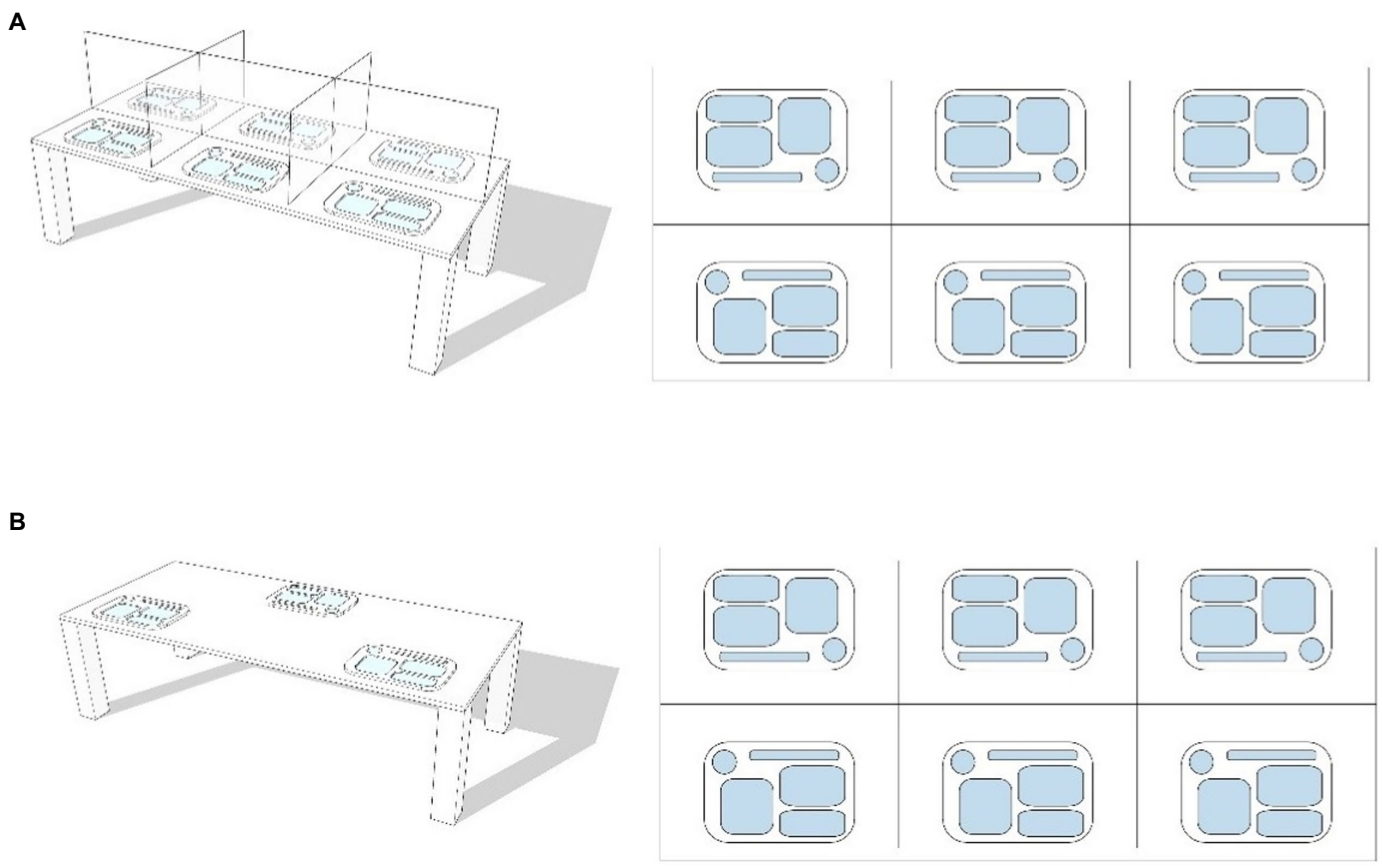
Schematic diagram of dining mode. **(A)** dining with baffle and **(B)** Dining across a seat.

The survey was conducted in the dining rooms of four elderly care facilities in four cities in northeast China (Harbin, Changchun, Shenyang, and Dalian). After the outbreak of the COVID-19, many elderly care facilities gradually changed their dining modes. The dining rooms in elderly care facilities in northeast China began to change the dining modes after June 2020. The dining rooms of these four elderly care facilities have adopted two dining modes: ‘dining with baffle’ and ‘dining across a seat’ (see Figure 1). These two modes are typical quarantine measures adopted by dining rooms in institutions with relatively dense populations during the COVID-19 pandemic in China. Even when the city was not under lockdown, the elderly care facilities were still under closed management and the dining mode continues to this day. The names, locations, scales, capacities, dining modes, and other information on the four elderly care facilities are shown in Table 1. Some surveys were conducted from April to June 2020, collecting data on normal dining modes; the survey and data collection for dining across a seat mode and dining with baffle mode were conducted from April to June 2021. During the period of complete closure and management of the elderly care facilities, the researchers could not or were allowed to enter, and part of the questionnaire was completed with the assistance of the staff. In elderly care facilities, the kitchen and dining room are either separated or connected (Mu and Kang, 2022). There are two kinds of tables: a round table and a table for four people. The four dining rooms considered in this study had four different layouts: the kitchen and dining room together with a round table layout; the kitchen and dining room connected with square tables for four people; independent dining room and round table layout; and an independent dining room with square tables for four people.

**Table 1:** Basic information on four elderly care facilities.

Name	City	Volume	Number of beds	Year of construction	Dining mode	Ownership	Price range (RMB/Month)
RF	Harbin	110,000	2000	2014	Dinning across a seat	Private	1800–4,500
AK	Harbin	66,000	1,500	2003	Dinning with baffle	Public	1800–3,500
JY	Changchun	1,600	60	2014	Dinning with baffle	Private	3,000–6,000
SHF	Changchun	15,000	300	1948	Dinning across a seat	Public	1,000–3,000

### 2.2. Participants

The elderly participants were asked to complete the survey and provide background information, such as age, gender, education level, and their satisfaction with the Indoor Environment Quality (IEQ) indicators of the dining rooms. The final analysis examined only the surveys completed by residents who had lived in the elderly care facility where the dining room was located for 6 months or more. We used Rockwood et al. (2005) frailty scale to evaluate the residents. A score of 1 indicates very good health, a score of 2 indicates good health, a score of 3 indicates good comorbidity after treatment, and a score of 4 indicates significant frailty. In general, older people who score between 1 and 4 are in good health. Elderly residents who scored between 1 and 4 on Rockwood et al. (2005) frailty scale were eligible for the study’s survey, meaning that they were physically and mentally fit enough to participate. According to the frailty scale, the elderly selected in this study were healthy and independent. Overall, 318 surveys were collected before the start of the epidemic, of which 153 were from males (48.1%) and 165 were from females (51.9%). After the start of the epidemic, 376 surveys were collected, of which 184 were from males (48.9%) and 192 were from females (51.1%). Details on the respondents of both surveys such as age, education level, and length of residence are shown in Table 2.

**Table 2:** Basic information about participants before COVID-19 outbreak.

Social characteristics	Classification	Number	Number
		(Before)	(After)
Gender	Male	153	184
	Female	165	192
	60–70	102	83
Age range (years)	71–80	78	103
	81–90	117	164
	≥90	21	26
	Primary school or lower	54	63
Education level	Junior school or senior school	155	181
	College or higher	109	132
Residence duration	<1 year	63	59
	1–3 years	95	82
	3–5 years	92	138
	>5 years	68	97
Dining Mode	Normal dining	318	204
	Dining across a seat	**-**	**-**
	Dining with baffle	**-**	172

### 2.3. Indoor environmental measurements of dining rooms

The study measured eight main environmental parameters of the dining rooms: SPL, luminance and illuminance levels, temperature, relative humidity, CO, CO_2_, and O_2_. The physical environment test consisted of two parts: a continuous test at three fixed test sites, and an immediate test at the end of the questionnaire.

Continuous tests were carried out in the four dining rooms used the measurement methods referred to in a previous joint study (Mu and Kang, 2022). Thermal environment, air temperature, and relative humidity were measured using a Centre 314 temperature/humidity data logger. Illumination and luminance were measured with a T-10A illuminometer and GPH-1001 luminance material as parameters to evaluate the light environment. SPL was used as a parameter to evaluate the sound environment and was recorded with 801 sound level meters. Indoor CO, CO_2_, and O_2_ concentrations were measured using a calibrated rapid response digital instrument (MS500-5). Air quality was analyzed using portable instruments.

The indoor environment of a dining room includes smells, such as the smell of frying and disinfection/sterilization materials required amid the COVID-19 pandemic. The human sense of smell is the most sensitive tool for evaluating and distinguishing odors and adapting to the environment (Henshaw, 2013). Therefore, this study measured the odour environment of the dining rooms subjectively rather than with an instrumental test. The dining modes and test spots of the four facilities are shown in Supplementary material.

### 2.4. Participant behavior observation

Image recording is a useful research method for collecting behavioral observations in older adults (Asan and Montague, 2014). In this study, a combination of photography and note-taking was used to record changes in the dining behavior of the elderly under different dining modes to investigate how the dining mode affected dining behavior without influencing the residents’ normal activities (Meng et al., 2017). During the dining periods, the researchers chose the best shooting position to eliminate blind spots in the corridor on the second floor of the dining room and took a photo every minute to record the dining time of the elderly and the number of people who communicated with each other while eating. According to the eating habits of the elderly, the behavior of the elderly was observed during lunch time. Combined with the opening hours of the restaurant, observations were made three times a week for one and a half hours each time from 11 am to 12.30 PM during the two survey periods.

### 2.5. Questionnaire survey

A questionnaire was used to explore the subjective perceptions and comfort levels of the elderly care facility residents regarding the acoustics, lighting, thermal comfort, air quality, odor environment, and overall environmental quality of the dining rooms. The survey comprised two parts. The first solicited background information on the participants, such as age, gender, and education level. The second part asked questions on subjective comfort levels with the dining rooms’ IEQ. The questionnaire was administered to the participants after observing them leaving the dining rooms at the end of the meal. During the survey, the participants stayed in the dining rooms for at least 15 min, after which they were interviewed face-to-face. A Likert Semantic Difference scale was used for the questionnaire. The participants evaluated their dining experience by selecting the appropriate words from among seven options: ‘very uncomfortable,’ ‘uncomfortable,’ ‘slightly uncomfortable,’ ‘neither comfortable nor uncomfortable,’ ‘slightly comfortable,’ ‘comfortable,’ and ‘very comfortable.’

### 2.6. Statistical analysis

SPSS 20.0 was used for statistical analysis. Pearson correlation analysis and linear regression analysis was used to determine the correlation between the respondents’ dining comfort evaluation and physical environmental factors, as well as the influence of the participants’ personal data on the evaluation results.

## 3. Results and discussion

### 3.1. Measurement data on dining rooms’ physical environment after the outbreak of COVID-19 pandemic

**Figure 2:**
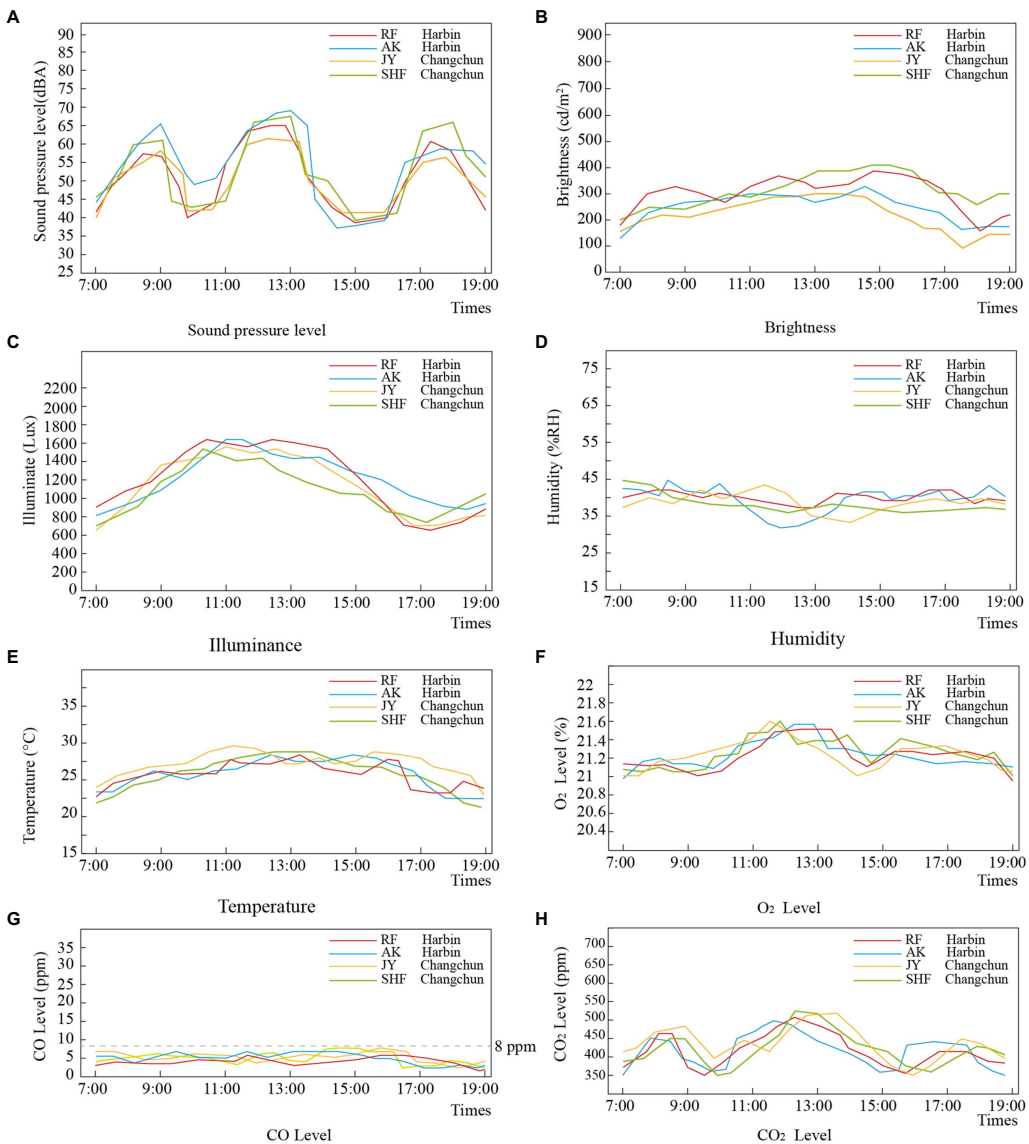
Measurement data of physical environment parameters of four dining rooms. **(A)** Sound pressure level, **(B)** brightness, **(C)** illuminance, **(D)** humidity, **(E)** temperature, **(F)** O_2_ Level, **(G)** CO Level, and **(H)** CO_2_ Level.

As indicated in Figure 2, the SPL of the dining rooms shows regular changes, with high values between 8:00 and 9:00, 11:00 to 13:00, and 17:00 to 18:00, when the SPL ranges from 40 to 70 dBA. This is consistent with the change periods noted in the physical environment survey conducted prior to the COVID-19 pandemic, but the peak is 10 decibels higher (Mu and Kang, 2022) during the COVID-19 outbreak. This may be because older people are more excited and have more conversations at mealtimes due to their limited outdoor activities. The brightness of the dining rooms ranges from 100 to 400 cd/m2, with a peak between 10:00 am and 15:00 pm. The illuminance ranges from 600 to 1,600 lux, and the peak occurs between 9:00 and 11:00. The brightness and illumination ranges do not differ between the pre-and post-pandemic periods. The humidity range is from 30 to 45%, which is higher than the 20 to 40% range before the COVID-19 period, and the valleys occur between 11:00 and 13:00. The temperature ranges from 23 to 28 degrees, with peaks at 11:00, 13:00, and 16:00, which is higher than the 20–25 degree range before the COVID-19 period. The O_2_ concentration in the dining rooms ranges between 21 and 21.6%, and usually peaks between 10:00 and 12:00. The concentration levels do not differ from the pre-COVID-19 levels, but the peak appears 2 h earlier. The CO_2_ levels range from 350 to 500 ppm and peak between 11:00 and 12:00, which is unchanged from the pre-COVID-19 levels. The CO levels are less than 8 ppm and peak between 14:00 pm and 16:00 pm; these levels are lower than the pre-COVID-19 level of 12 ppm. The changes in humidity, temperature and air quality are linked to the widespread belief that central air-conditioning systems would accelerate the spread of COVID-19 (Chirico et al., 2020) and their consequent reduction or elimination after the outbreak, along with a corresponding increase in natural ventilation frequency.

### 3.2. Effects of dining rooms’ physical environment on dining comfort of elderly before and after the start of the COVID-19 pandemic

#### 3.2.1. Subjective comfort level of dining rooms’ physical environment

**Figure 3:**
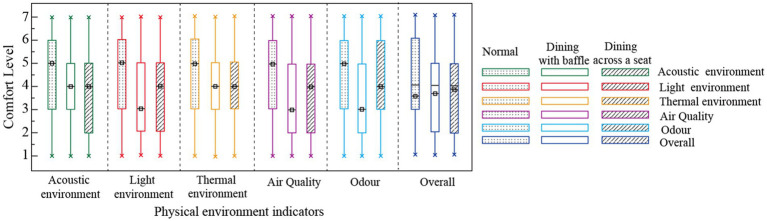
Comfort evaluation of dining room environment before and after start of COVID-19 pandemic.

The study found that changes in dietary patterns can affect IEQ satisfaction. The COVID-19 pandemic has changed dining modes and affected user satisfaction (Chirico et al., 2020), including for the elderly living in care facilities. In the normal dining mode before the outbreak of the COVID-19 pandemic, the elderly’s overall evaluation of the dining rooms’ acoustic environment, light environment (including brightness and illuminance), thermal environment (including temperature and humidity), air quality (i.e., ventilation), and odor was high (with all more than 5 points out of 7; the scores are shown in Figure 3). After the outbreak of the COVID-19 pandemic, ventilation frequency in buildings increased (Sun and Zhai, 2020) due to the need for epidemic prevention, which affected indoor temperature, humidity, and air quality and thus comfort levels. In the ‘dining across a seat’ mode, the respondents gave their subjective comfort with the acoustic environment, thermal environment, air quality, and odor environment a 4-point score, which was lower than the normal dining mode score. In the ‘dining with baffle’ mode, the thermal environment was scored at 4 points, but the sound environment, light environment, air quality, and odor environment were all given 3 points, which is lower than the score for the dining across a seat mode. This result is in line with the research finding that people prefer to eat at a safe distance rather than in isolation in regular dining rooms (Wang et al., 2021). The dining with a baffle mode and dining across a seat mode adopted by dining rooms in elderly care facilities after the outbreak of the COVID-19 pandemic have indeed affected the dining comfort of the residents. In this study, the lowest subjective comfort level score was given to the dining with baffle mode.

The scores for several physical environmental factors were lower in the dining with baffle mode and across a seat mode than for the normal dining mode (see Table 3), indicating that the changes in dining mode caused by the COVID-19 pandemic have affected the dining comfort of the elderly. The baffle mode was rated highest on IEQ, scoring nearly 5 on acoustic, air quality, and odor quality (*p* < 0.01). The participants generally reported more comfort in the dining across a seat mode than the baffle mode. The evaluation of acoustic (4.859) and odor (4.748) quality was significantly higher for the baffle mode than for the dining across a seat mode (3.712 and 3.752 respectively), and the evaluation of the thermal environment (5.237) was much higher for the dining across a seat mode than for the baffle mode (3.881; *p* < 0.01). The comfort level of the thermal environment may be higher in the dining across a seat mode because that mode reduces the number of people who are in the dining room at the same time, making the temperature and humidity more comfortable. Thus, the environmental requirements of the elderly can be met in dining rooms in terms of sound, light, temperature, and air quality more easily than is possible in other facility spaces where the elderly stay longer, such as bedrooms and activity halls (Mu and Kang, 2022). Environmental requirements for dining rooms are often ignored or not satisfied (Wu and Lin, 2022). In elderly care facilities, dining rooms are often noisy and overstimulating places, and mealtime activities are important to seniors. Inappropriate physical environments in dining rooms represent one of the most frequently expressed concerns among elderly care facility staff (Chaudhury et al., 2013). The need for changes in dining modes during the COVID-19 isolation period require dining rooms in elderly care facilities to design their physical environments in a way that improves dining comfort for the elderly.

**Table 3:** Evaluation of IEQ factors in dining rooms based on differences in dining modes.

	Factors	Acoustic environment	Lighting environment	Thermal environment	Air quality	Odor environment
Factors of dining mode	Normal	5.013	4.102	5.074	4.862	4.983
	Dining with baffle	3.712	4.027	5.237	4.179	3.752
	Dining across a seat	4.859	4.136	3.881	4.723	4.748
	*F*	52.673	43.930	41.512	42.732	43.930
	*P*	0	0.035	0	0	0
	$\eta_{p}^{2}$	1.612	1.8263	1.412	1.504	1.249

#### 3.2.2. Correlation of measured dining rooms physical environment with subjective comfort level results

**Figure 4:**
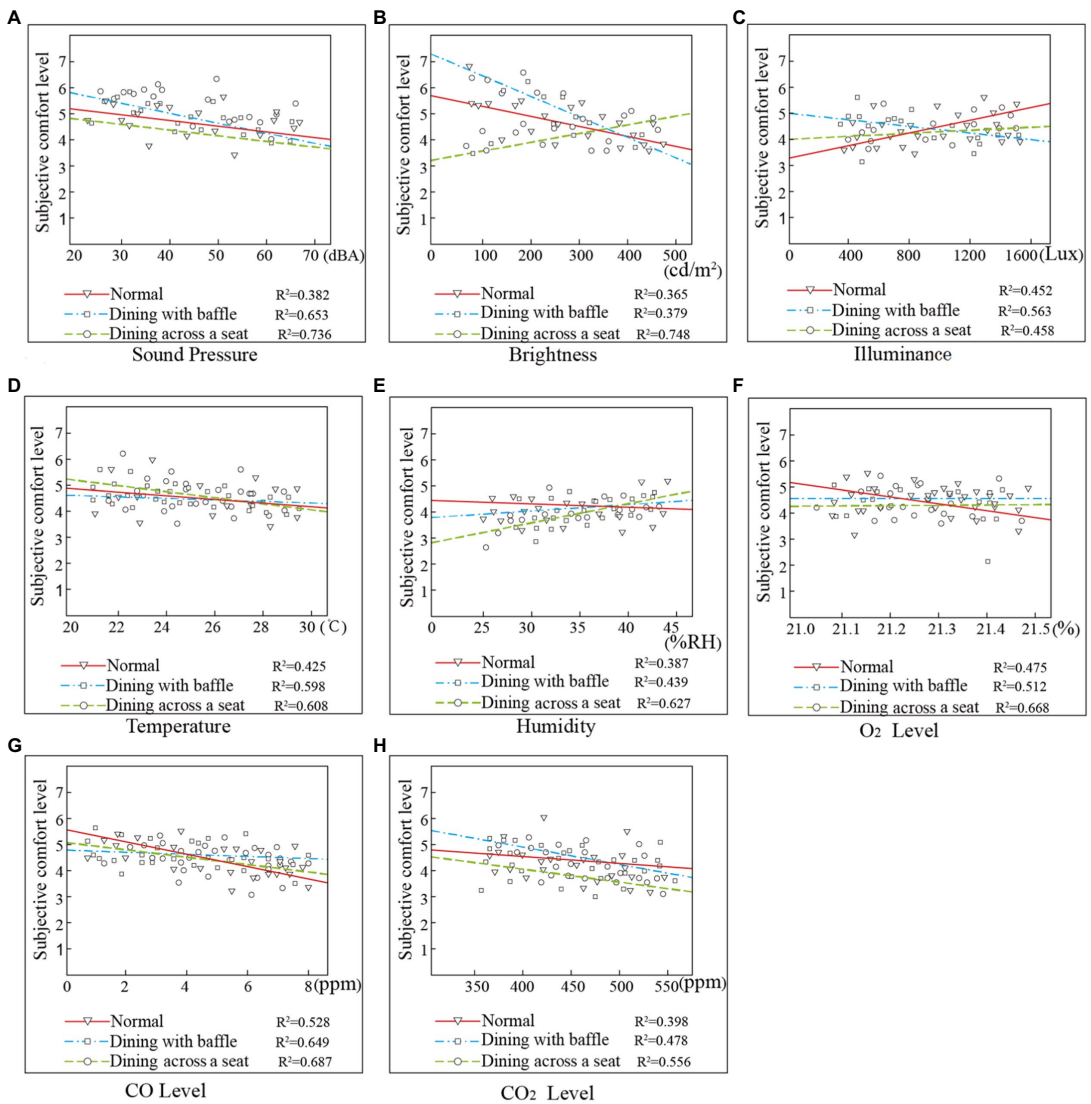
Data fitting of results for measured values and subjective comfort level results. **(A)** SPL, **(B)** illuminance, **(C)** brightness, **(D)** temperature, **(E)** humidity, **(F)** O_2_ Level, **(G)** CO Level, and **(H)** CO_2_ Level.

As Figure 4A shows, the linear regression effect of the SPL value and the evaluation results for dining with a baffle and dining across a seat is good, with *R*^2^ values of 0.653 and 0.736, respectively, both exceeding 0.5. The *R*^2^ value of the normal dining mode is 0.382, and the linear regression effect is poor. This indicates that the elderly are more sensitive to the acoustic environment in the dining modes after the outbreak of the COVID-19 pandemic. The various isolation measures taken during the COVID-19 pandemic have reduced noise, but this constitutes passive noise reduction in a threatened environment, which creates discomfort and reduces noise tolerance (Caniato et al., 2021; Ferrer-Torres and Gimenez-Llort, 2021).

Figure 4B shows that, for brightness, the fitting effect of normal dining and dining with a baffle is poor, not exceeding 0.5. The fitting effect of dining with a baffle is better. For illuminance (Figure 4C), the fitting effect is poor, and neither the normal mode nor dining across a seat mode exceeds 0.5. This shows that the dining mode has less influence on the subjective comfort level with the lighting environment for the elderly, and only the dining across a seat mode has a greater impact on the participants’ subjective comfort level with brightness. As people age, the efficiency of their acceptance of outside information declines; the elderly are also more prone to feel lonely, artificial personal distancing tends to alienate them (Mokros and Deetz, 2013), and they are more strongly affected by the light environment in dining rooms (Stroebele and De Castro, 2004). Therefore, it is necessary to strengthen the design of lighting environments in the dining rooms of elderly care facilities during the COVID-19 pandemic to improve the residents’ dining comfort.

Figure 4D shows that, for temperature, the R^2^ value fitted by the measured value and the subjective comfort level is 0.598 in the dining with baffle mode and 0.608 in the dining across a seat mode; both are higher than the normal dining mode values. For humidity (see Figure 4E), the *R*^2^ value fitted by the measured value and the comfort evaluation is 0.627 in the dining across a seat mode, indicating that the residents’ evaluation of the thermal environment is affected by temperature and humidity most strongly in the dining across a seat mode.

As shown in Figures 4F–H, the fitted value between air quality and the residents’ comfort evaluation is highest in the dining across a seat mode, where the *R*^2^ values of O_2_ and CO exceed 0.6. This indicates that the air quality is higher in the dining across a seat mode than in the dining with baffle mode, perhaps because fewer people are seated together at the same time in the dining across a seat mode.

### 3.3. Influence of different dining modes on elderly before and after start of COVID-19 pandemic

#### 3.3.1. Influence of different dining modes on dining comfort of elderly

Dining comfort is an important component of quality of life in elderly care facilities, and measuring and improving dining comfort may be an effective way to improve quality of life among the elderly (Pizzola et al., 2013). The comfort felt by the elderly while dining can be observed in their behavior during the dining process. In this study, we observed changes in dining time, dining crowd density, and communication frequency among the participants under different dining modes.

**Figure 5:**
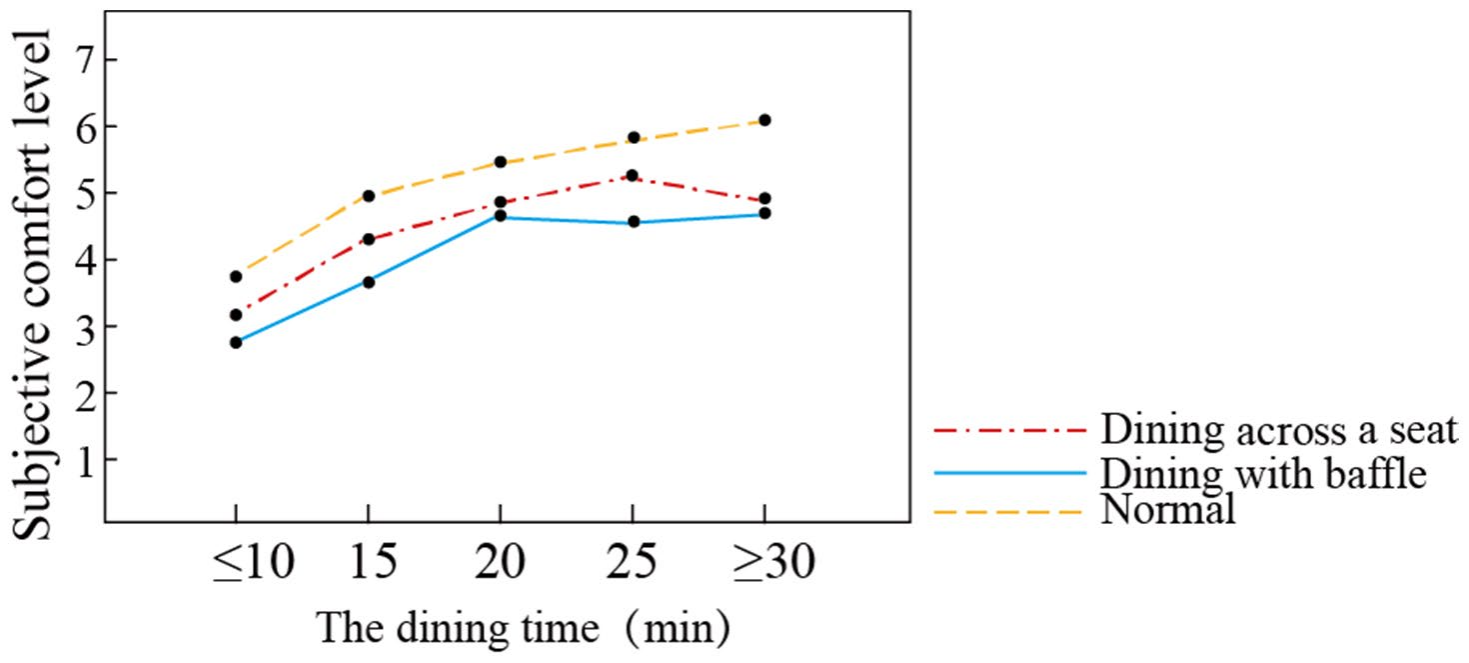
Dining time of elderly in different dining modes.

Interventions or interference during dining room use can lead to changes in meal times, which in turn can affect dining comfort and eating status in older adults (Keller et al., 2014). We found that dining comfort values under each dining mode differed according to dining time (see Figure 5). In the normal dining mode, the participants’ subjective comfort level increased as their dining time increased. In the dining across a seat mode, subjective comfort level is proportional to the dining time during the 10–25 min dining period, but their subjective comfort level decreases during the 25- and 30-min dining periods. In the dining with baffle mode, the subjective comfort level increases during the 10–20 min dining period and reaches a peak at 20 min; the level decreases during the 20–30 min dining period. Thus, the dining with baffle mode and across a seat mode have both affected the subjective dining comfort level of the elderly. In both modes, the elderly tend to end their meals faster; the dining across a seat mode has a slightly lower impact than the dining with baffle mode. The research indicates that maintaining an enjoyable diet for as long as possible is one of the determinants of promoting good nutrition in an aging population (Bailly et al., 2020). Thus, ways to improve the dining comfort of seniors in elderly care facilities during COVID-19 isolation warrants further research. Several studies have shown that increasing changes in single contextual elements during dining periods, such as adding background music or table decorations, can affect food intake in older adults (Divert et al., 2015; Gu et al., 2021). Therefore, various methods can be used to reduce the emotional pressure on the elderly and improve their comfort in dining rooms that adopt abnormal dining modes during the COVID-19 isolation period, such as by improving the dining room setting.

**Figure 6:**
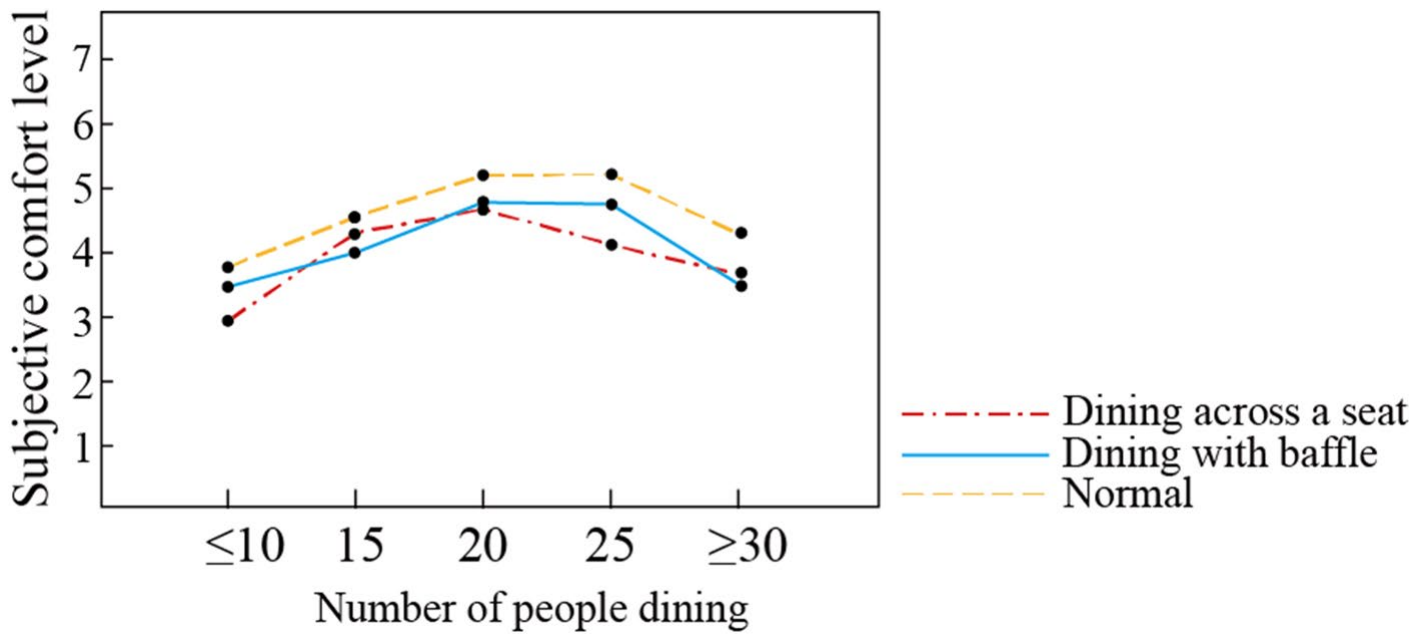
Dining number of elderly in different dining modes.

Differences were observed in crowd density and comfort evaluation values under different dining modes, as shown in Figure 6. The participants’ comfort evaluation was lower in the dining with baffle mode and dining across a seat mode than in the normal dining mode. In the normal dining and dining with baffle modes, the subjective comfort level was highest for 25 people in the dining room, while this level was highest for 20 people in the dining across a seat mode. The subjective comfort level for 15 people was higher in the dining across a seat mode than in the dining with baffle mode. The comfort score is low in both modes (4 points or less), and the corresponding subjective comfort level is ‘neither uncomfortable nor comfortable.’ The number of people dining at the same time is reduced by half in the dining across a seat mode, and its overall subjective comfort level is higher than that of dining with baffle mode. In the dining with baffle mode, comfort level is affected most strongly by crowd density. Therefore, in the dining with baffle mode, the number of diners should be controlled, or the meals should be served in batches.

**Figure 7:**
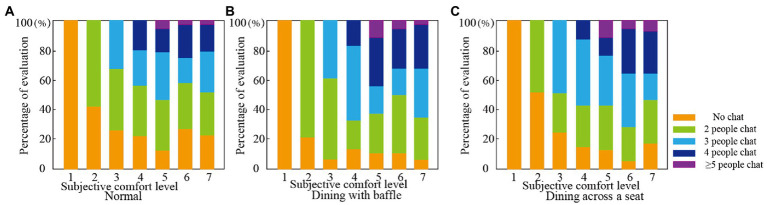
Communication frequency of elderly in different dining modes. **(A)** Normal, **(B)** dining with baffle, and **(C)** dining across a seat.

Mealtime is a significant social activity during which older adults interact (Curle and Keller, 2010). Different dining modes have distinct effects on the comfort of the elderly, which can be observed in their dining behavior, specifically the frequency of communication during dining. In general, people are more willing to talk to others when they are in a good mood, and frequency of communication is generally proportional to the pleasure experienced during dining (Meng et al., 2017). Studies have also shown that silence during meals has adverse effects on the eating habits and health of seniors in elderly care facilities (Sidenvall, 1999). Given the differences in scale between the dining rooms surveyed in this study, the proportion of the number of people talking during dining periods out of the total number of people dining was used as a measure of communication frequency. The communication frequency levels of the participants in three dining modes (normal, dining with baffle, and dining across a seat) are shown in Figure 7. In the normal dining mode, most high comfort evaluation scores were given for no conversation and two-person conversations, while the fewest high scores were given for conversations with more than three people. This indicates that, in the normal dining mode, the elderly tend to have conversations but also have certain requirements for quiet time. After the start of the COVID-19 pandemic, the proportion of high scores for no conversation and two-person conversations decreased, while the proportion of high scores for more than three people talking increased. In the dining across a seat mode, the proportion of high comfort scores for no conversation and two-person conversation is lower than that for the normal dining mode, and the proportion of high scores for conversations with more than three people is higher than that in the normal dining mode. Thus, the changes in dining modes in elderly care facilities due to the COVID-19 pandemic have affected frequency of communication, with the highest comfort score given to multi-person communication in the dining across a seat mode. The comfort rating for multi-person conversation is also higher in the dining with baffle mode than in the normal mode, perhaps because the baffle can isolate and absorb part of the noise and give the elderly a sense of security.

#### 3.3.2. Correlation between physical environment and crowd density in different dining modes

**Figure 8:**
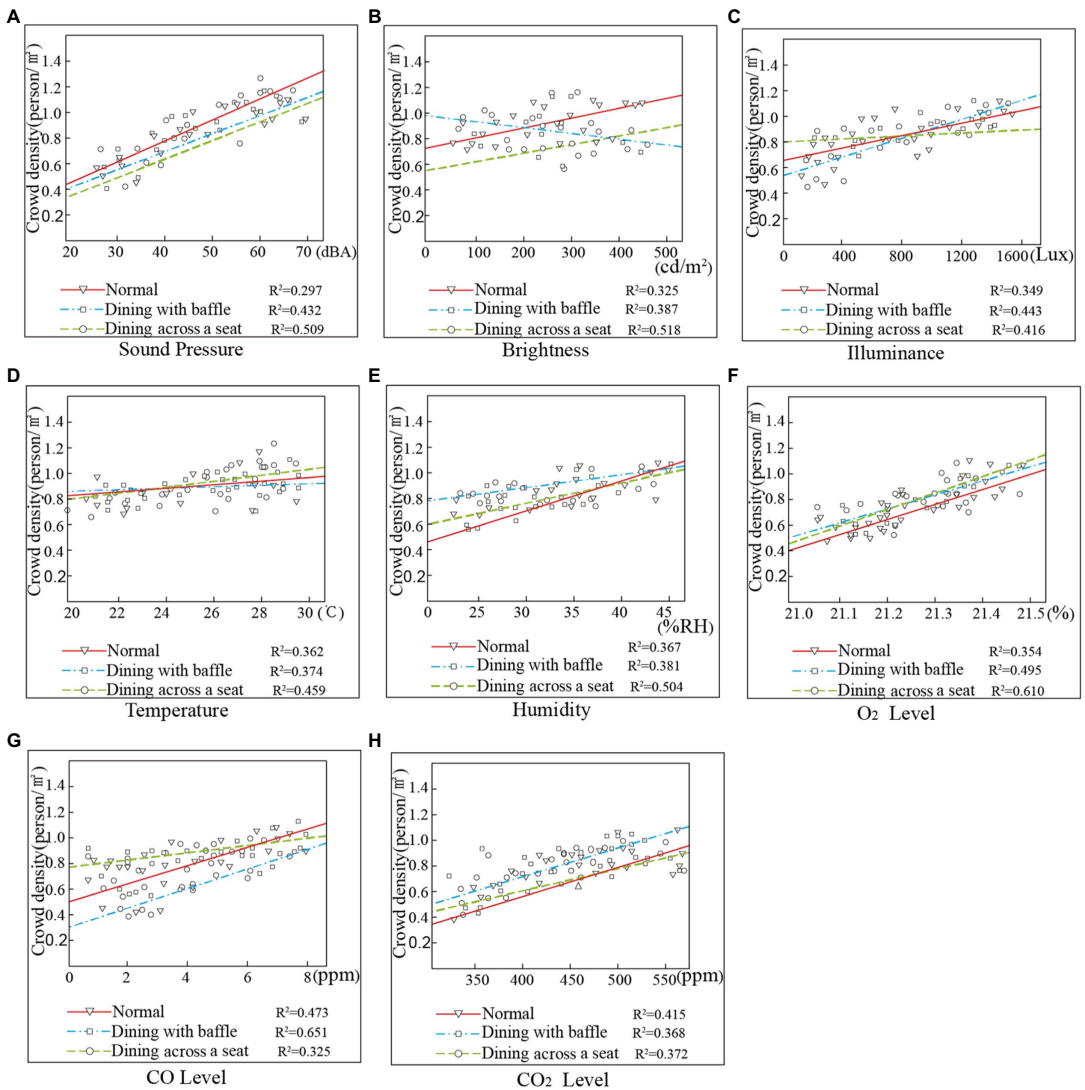
Data fitting of environmental measurements and crowd density. **(A)** SPL, **(B)** illuminance, **(C)** brightness, **(D)** temperature, **(E)** humidity, **(F)** O_2_ Level, **(G)** CO Level, and **(H)** CO_2_ Level.

The correlations between the physical environment values and crowd density are shown in Figure 8. In terms of SPL (see Figure 8A), the linear regression effect of the measured value and crowd density for the dining with baffle and dining across a seat modes is good. The *R*^2^ value of the normal dining mode is 0.297, and the linear regression effect is poor. Studies have shown that older adults rate acoustic environmental comfort more highly and have a greater tolerance for high-decibel sound as they age (Cui et al., 2021). The elderly living in care facilities usually need assistance from others, and social distancing restrictions are a problem for them (Goggin and Ellis, 2020). Therefore, when dining in rooms under COVID-19-related isolation measures, they will be more inclined to gather to help each other, which is consistent with the results of this study.

In the linear regression of the brightness value and the result for crowd density, the *R*^2^ value of the dining across a seat mode is 0.518 (see Figure 8B). Under identical brightness conditions, the elderly are more inclined to gather in the dining with baffle mode. The *R*^2^ values of the linear regression of the illuminance and crowd density values for the three dining modes (see Figure 8C) show that the dining mode has little effect on crowd density among the elderly.

In terms of temperature (see Figure 8D), the linear regression *R*^2^ values of the temperature and crowd density values for the three dining modes were 0.362, 0.374, and 0.459; the elderly had a higher temperature tolerance in the dining across a seat mode. This may be because the dining room accommodates fewer people eating at the same time in this mode, and greater density leads to higher temperatures. In terms of humidity (see Figure 8E), the linear regression *R*^2^ value of the humidity and crowd density values in the dining across a seat mode is 0.504, indicating that the elderly have a higher tolerance to humidity in the dining across a seat mode. This may also be because fewer people are eating at the same time in this mode.

Regarding O_2_ (see Figure 8F), the crowd density and measured values had a higher degree of fit in the dining across a seat mode, with an *R*^2^ value of 0.610; this shows that the elderly need more oxygen in the dining with baffle mode. In terms of CO, the linear regression *R*^2^ value of the measured value and crowd density value in the dining with baffle mode is 0.651 (see Figure 8G); this shows that crowd density is most affected by CO levels in this mode, perhaps because the installation of baffles impedes air circulation. This result is consistent with studies showing that air quality in dining rooms is acceptable to people (Cheung et al., 2017). The isolation measures implemented in dining rooms have had a greater impact on the elderly, and more attention should be paid to the improvement of air quality in the dining with baffle mode.

### 3.4. Influence of demographic factors on dining comfort in different dining modes

Due to differences in age and physical fitness levels, the dining habits of the elderly are quite different from those of the young; for example, the former take longer to eat their meals and need brighter lights. The elderly also have a higher risk perceptions amid the COVID-19 pandemic than other groups, and they have a greater fear of using public places (Fabisiak et al., 2020). Dining comfort is a subjective evaluation that can be greatly affected by background factors, including physiological factors such as gender and age; social factors such as education level and marital status; and lifestyle factors such as residence duration and place of origin. This section analyses the influence of these demographic factors on the dining comfort of the elderly participants based on the survey results. Age, education level, and residence duration were considered in the analysis, as these individual and social factors may affect dining comfort.

#### 3.4.1. Influence of age on evaluation of dining comfort in different dining modes

This study examined differences in participants’ subjective comfort evaluation of acoustics, lighting, thermal environment, air quality, and odor by age group in different dinning modes before and after the outbreak of the COVID-19 pandemic.

**Figure 9:**
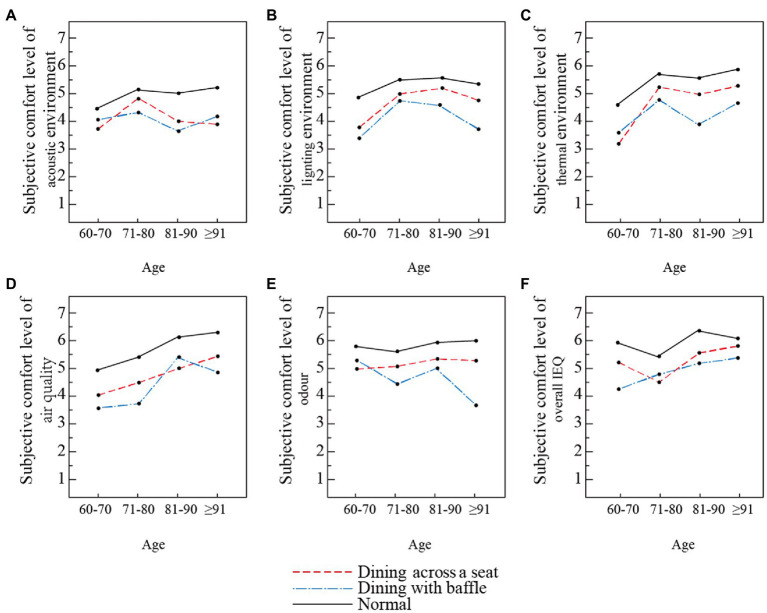
Comfort evaluation of dining rooms’ physical environment based on age in three dining modes. **(A)** Acoustic environment, **(B)** light environment, **(C)** thermal environment, **(D)** air quality, **(E)** odor environment, and **(F)** overall IEQ.

For the participants of all ages, the subjective comfort levels of the three dining modes are ranked as follows: normal dining > dining with baffle > dining across a seat (see Figure 9). The subjective comfort levels for the light environment and odor environment in the dining with baffle mode is much lower for those over 90 years old than for other age groups, as shown in Figures 9B,E. Lighting and odor environments are important physical environments in a dining room. Among the factors that have strong impacts on dining comfort (Stroebele and De Castro, 2004), the subjective comfort level observed in the dining with baffle mode is much lower than that of other dining modes.

#### 3.4.2. Correlation between measurements and personnel density

**Figure 10:**
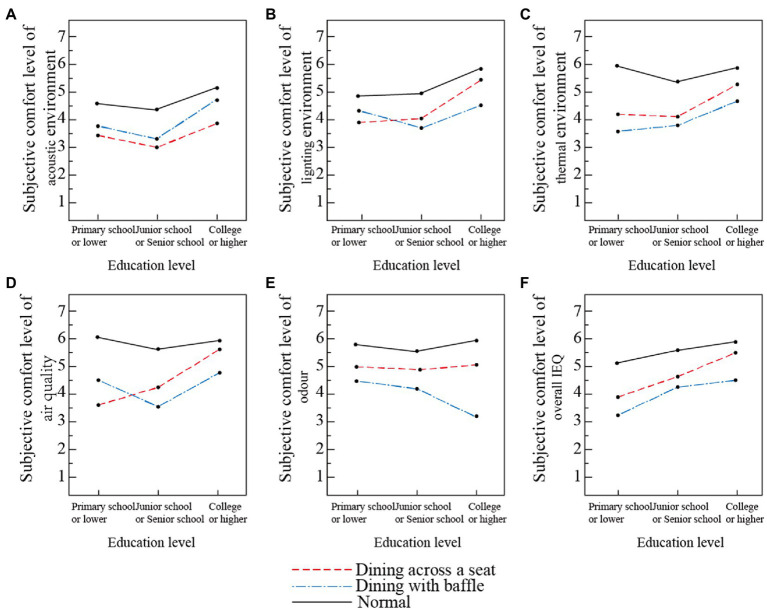
Comfort evaluation of dining rooms’ physical environment based on education level in three dining modes. **(A)** Acoustic environment, **(B)** light environment, **(C)** thermal environment, **(D)** air quality, **(E)** odor environment, and **(F)** overall IEQ.

The subjective comfort levels differ among the participants according to education level in the different dining modes (see Figure 10). In general (aside from acoustic environment), the physical environment comfort evaluations in all three dining modes are ranked as follows: normal dining > dining across a seat > dining with baffle. For the acoustic environment, dining across a seat is scored higher than dining with baffle, perhaps due to the inconvenience of communication and low communication frequency in that mode. The participants with primary education and below and those with college education and above gave higher evaluations of the acoustic environment, light environment, thermal environment, and air quality, while participants with middle school education gave lower evaluations. Older people with higher education levels gave lower evaluations of the odor environment, perhaps because those with higher education levels use more detailed and precise classifications in their evaluation of smell (Mullol et al., 2012). Moreover, the higher the education level, the higher was the participants’ acceptance of dining across a seat. For participants with education levels below primary school, their light environment and air quality evaluations were higher for the dining with baffle mode than for the dining across a seat mode. For both the normal dining mode and dining across a seat mode, the participants’ evaluations of the overall environment were directly proportional to their education level, with the highest overall comfort evaluation given by those with middle school education to the dining with baffle mode.

#### 3.4.3. Influence of residence duration on evaluation of dining comfort in different dining modes

**Figure 11:**
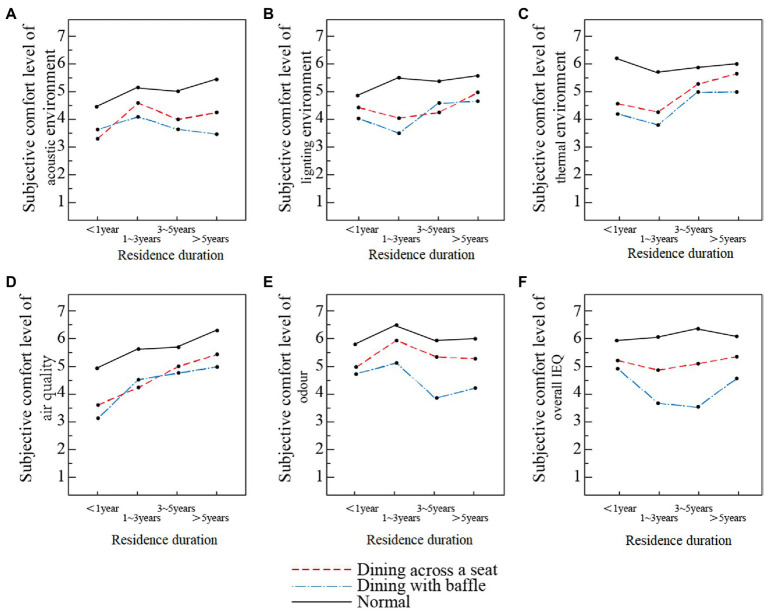
Comfort evaluation of dining rooms’ physical environment based on residence duration in three dining modes. **(A)** Acoustic environment, **(B)** light environment, **(C)** thermal environment, **(D)** air quality, **(E)** odor environment, and **(F)** overall IEQ.

Overall, the subjective comfort levels of the dining rooms’ physical environments in the three dining modes were ranked as follows: normal dining > dining across a seat > dining with baffle (see Figure 11). In the normal dining mode, residence duration had no effect on the evaluation of overall environmental comfort. For both dining with baffle mode and dining across a seat mode, participants who had lived at the facility for less than 1 year or more than 5 years gave higher scores, while those who had lived there between 1 and 3 years or from 3 to 5 years gave lower scores. In the dining with baffle mode, participants who had lived at the facility for 3–5 years gave the lowest evaluation on overall IEQ comfort. In general, evaluations of the living environment increase as the time spent in elderly care facilities increases (Stuck et al., 1995). We found that changes of dining mode significantly affected the dining experience of the elderly participants.

## 4. Conclusion

This study examined how changes in dining room environment and dining mode affect the dining comfort of the elderly in four elderly care facilities in northeast China that adopted isolation measures during the COVID-19 pandemic period. Our analysis shows that changes of dining mode affect the comfort of the elderly. The main conclusions of this study are as follows.

The physical environmental parameters of the four dining rooms differed from those before the epidemic, as shown by the increased sound pressure, humidity, and temperature levels. The SPL peak was 10 decibels higher. The humidity range was 30–45%, higher than that before the COVID-19 crisis. The temperatures ranged from 23 to 28 degrees, which were higher than the levels before the COVID-19 period. The oxygen concentration in the dining room did not change, but the peak time appeared 2 h earlier. The carbon monoxide levels were lower than the pre-COVID-19 levels, while the lighting environment parameters and carbon dioxide levels were unchanged from the pre-COVID-19 levels. The changes in humidity, temperature and air quality are linked to the widespread belief that central air-conditioning systems would accelerate the spread of COVID-19 and their consequent reduction or elimination of their use after the outbreak, along with a corresponding increase in natural ventilation frequency.

The changes in the physical environments of dining rooms after the outbreak of the COVID-19 pandemic have affected the dining comfort of the elderly. In the normal dining mode, participants rated the subjective comfort level of the physical environment at 5 points, while the dining across a seat mode was given 4–5 points, and the dining with baffle mode received 3–4 points. The dining comfort of the elderly in the dining with baffle mode is lower than that in the dining across a seat mode. For thermal environment, the correlation fit was better in the dining across a seat mode, indicating that the participants’ evaluations of the thermal environment were affected by the objective conditions of temperature and humidity most strongly in the dining across a seat mode. For air quality, the fitting effect between the air quality data and comfort evaluation values was best in the dining across a seat mode, indicating that the elderly participants were most susceptible to dining room air quality conditions in that mode. This result shows that the elderly prefer to eat at a safe distance rather than in isolation in dining rooms. The need for changes in dining modes during the COVID-19 isolation period require dining rooms in elderly care facilities to design their physical environments in a way that improves dining comfort for the elderly.

The dining behavior of the participants was also affected by the dining mode, including dining time, frequency of communication, and crowd density. The elderly tended to finish their meals faster than they did in the normal dining mode, with the dining across a seat mode influenced by the change slightly less than the dining with baffle mode. The proportion of participants favoring multi-person communication was higher than that in the normal dining mode, but the subjective comfort level was lower. Crowd density had the greatest impact on dining comfort in the dining with baffle mode. The SPL tolerance of the participants was stronger in the dining with baffle mode and dining across a seat mode, and the thermal environment was rated as being more comfortable in the dining across a seat mode. Lighting environment and air quality were found to have little effect on crowd density. Various methods can be used to reduce the emotional pressure on the elderly and improve their comfort in dining rooms that adopt abnormal dining modes during the COVID-19 isolation period, such as by improving the dining room setting.

In terms of demographic factors, the participants’ age, education level, and length of residence were found to be the main influencing factors in dining comfort for all dining modes. Overall, participants of all ages, education levels, and lengths of residence ranked the comfort of the dining rooms’ physical environment as follows: normal dining > dining across a seat > dining with baffle. Participants aged 71–80 years old rated the comfort level of dining with baffle mode higher than that in the dining across a seat mode. The higher the participants’ education level, the higher their acceptance of dining across a seat. In the dining with baffle mode, participants who had lived at the residence for 3–5 years gave the lowest evaluation on overall IEQ comfort. The impact of demographic factors on the evaluation results also requires elderly care facilities to adjust the dining style in time according to the characteristics of different groups, so as to ensure that the elderly are comfortable when isolation measures may be adopted in the restaurant for other reasons in the future.

This study examined the environment of dining rooms in four elderly care facilities in China during the COVID-19 pandemic. The data were statistically analyzed using site-specific parameters and subjective surveys of older adults, which leads to several limitations. This study investigated only the subjective evaluations of elderly respondents on the comfort levels of the environment in dining rooms in elderly care facilities. It did not consider other factors that may affect subjective comfort levels in the dining experience, such as food and service quality. Healthy dining, including the quality of the dining environment, is very important for the elderly. Quality of life in tightly clustered elderly care facilities needs to be monitored closely, given the susceptibility of the elderly population to COVID-19 infection. This study’s comparative analysis of how the environmental features of the dining with baffle mode and dining across a seat mode affect the elderly suggest that the acoustic environment and air quality of dining rooms in elderly care facilities should be optimized. The results of this study can be used to guide the optimal design of dining modes in the dining rooms of elderly care facilities under the requirements of reducing the infection rate during the epidemic of COVID-19 and similar diseases. It is also very important to study how dining modes influence the subjective comfort levels of the elderly in the context of COVID-19 prevention and control measures in order to improve the quality of life of residents in elderly care facilities.

## Data availability statement

The original contributions presented in the study are included in the article/Supplementary material, further inquiries can be directed to the corresponding author.

## Author contributions

JM and JK contributed to conception and design of the study and wrote sections of the manuscript. JM organized the database, performed the statistical analysis, and wrote the first draft of the manuscript. All authors contributed to the manuscript revision, read, and approved the submitted version.

## Funding

This study was supported by the Youth Program of National Natural Science Foundation of China (52208017); China Postdoctoral Science Foundation (2022M720959); China Association for Science and Technology Think Tank Young Talents Program 2021 (grant number: 2021ZZZLFZB1207149).

## Conflict of interest

The authors declare that the research was conducted in the absence of any commercial or financial relationships that could be construed as a potential conflict of interest.

## Publisher’s note

All claims expressed in this article are solely those of the authors and do not necessarily represent those of their affiliated organizations, or those of the publisher, the editors and the reviewers. Any product that may be evaluated in this article, or claim that may be made by its manufacturer, is not guaranteed or endorsed by the publisher.

## References

[ref1] Asan O. Montague E. (2014). Using video-based observation research methods in primary care health encounters to evaluate complex interactions. Inform. Prim. Care 21, 161–170. doi: 10.14236/jhi.v21i4.72, PMID: 25479346 PMC4350928

[ref2] BaillyN.Van WymelbekeV.MaîtreI.Sulmont-RosséC. (2020). The assessment of eating pleasure among older adults: development and preliminary validation of the anticipatory and consummatory eating pleasure (ACEPS). J. Nutr. Health and Aging 24, 606–613. doi: 10.1007/s12603-020-1388-2, PMID: 32510113

[ref3] Bakar A. Z. A. Ganesan L. Othman M. Haron S. A. Ishak F. A. C. (2020). Where to eat: exploring silver consumer restaurant dining choice in Malaysia. JSSH 28, 3297–3317. doi: 10.47836/pjssh.28.4.44

[ref4] Bloom I. Lawrence W. Barker M. Baird J. Dennison E. Sayer A. A.. (2017). What influences diet quality in older people? A qualitative study among community-dwelling older adults from the Hertfordshire cohort study, UK. Public Health Nutr. 20, 2685–2693. doi: 10.1017/S1368980017001203, PMID: 28724471 PMC5612401

[ref5] Caniato M. Bettarello F. Gasparella A. (2021). Indoor and outdoor noise changes due to the COVID-19 lockdown and their effects on individuals’ expectations and preferences. Sci. Rep. 11, 16533–16517. doi: 10.1038/s41598-021-96098-w, PMID: 34400713 PMC8368209

[ref6] Chaudhury H. Hung L. Badger M. (2013). The role of physical environment in supporting person-centered dining in long-term care: a review of the literature. Am. J. Alzheimer’s Dis. Other Dement. 28, 491–500. doi: 10.1177/1533317513488923, PMID: 23687182 PMC10852766

[ref7] Cheung T. C. Schiavon S. Gall E. T. Jin M. Nazaroff W. W. (2017). Longitudinal assessment of thermal and perceived air quality acceptability in relation to temperature, humidity, and CO2 exposure in Singapore. Build. Environ. 115, 80–90. doi: 10.1016/j.buildenv.2017.01.014

[ref8] Chirico F. Sacco A. Bragazzi N. L. Magnavita N. (2020). Can air-conditioning systems contribute to the spread of SARS/MERS/COVID-19 infection? Insights from a rapid review of the literature. Int. J. Environ. Res. Public Health 17:6052. doi: 10.3390/ijerph17176052 32825303 PMC7503634

[ref9] Comas-Herrera A. Ashcroft E. Lorenz-Dant K. (2020). “International examples of measures to prevent and manage COVID-19 outbreaks in residential care and nursing home settings,” in Report, LTCcovid org, International Long-Term Care Policy Network. eds. Comas-Herrera A. Ashcroft E. C. Lorenz-Dant K. (London: CPEC-LSE).

[ref10] Cori L. Bianchi F. Cadum E. Anthonj C. (2020). Risk perception and COVID-19. Int. J. Environ. Res. Public Health 17:3114. doi: 10.3390/ijerph17093114 32365710 PMC7246460

[ref11] Cui P. Zhang J. Li T. T. (2021). Research on acoustic environment in the building of nursing homes based on sound preference of the elderly people: a case study in Harbin, China. Front. Psychol. 12:707457. doi: 10.3389/fpsyg.2021.707457, PMID: 34744868 PMC8563576

[ref12] Curle L. Keller H. (2010). Resident interactions at mealtime: an exploratory study. Eur. J. Ageing 7, 189–200. doi: 10.1007/s10433-010-0156-2, PMID: 28798628 PMC5547351

[ref13] Damayanthi H. Prabani K. I. P. (2021). Nutritional determinants and COVID-19 outcomes of older patients with COVID-19: a systematic review. Arch. Gerontol. Geriatr. 95:104411. doi: 10.1016/j.archger.2021.104411, PMID: 33836322 PMC8010373

[ref14] Divert C. Laghmaoui R. Crema C. Issanchou S. Wymelbeke V. V. Sulmont-Rosse C. (2015). Improving meal context in nursing homes. Impact of four strategies on food intake and meal pleasure. Appetite 84, 139–147. doi: 10.1016/j.appet.2014.09.027, PMID: 25445198

[ref15] Fabisiak B. Jankowska A. Kłos R. (2020). Attitudes of polish seniors toward the use of public space during the first wave of the COVID-19 pandemic. Int. J. Environ. Res. Public Health 17:8885. doi: 10.3390/ijerph17238885, PMID: 33260396 PMC7729857

[ref16] Ferrer-Torres A. Gimenez-Llort L. (2021). Confinement and the hatred of sound in times of COVID-19: a Molotov cocktail for people with Misophonia. Front. Psych. 12:627044. doi: 10.3389/fpsyt.2021.627044, PMID: 34040551 PMC8141632

[ref17] Goggin G. Ellis K. (2020). Disability, communication, and life itself in the COVID-19 pandemic. Health Sociol. Rev. 29, 168–176. doi: 10.1080/14461242.2020.1784020 33411654

[ref18] Gu Q. Li M. Kim S. S. (2021). The role of nostalgia-evoking stimuli at nostalgia-themed restaurants in explaining benefits, consumption value and post-purchase behavioral intention. Int. J. Hosp. Manag. 96:102955. doi: 10.1016/j.ijhm.2021.102955

[ref19] Henshaw V. (2013). Urban Smellscapes: Understanding and Designing City Smell Environments, 1–256.

[ref20] Iaboni A. Cockburn A. Marcil M. Rodrigues K. Marshall C. Garcia M. A.. (2020). Achieving safe, effective, and compassionate quarantine or isolation of older adults with dementia in nursing homes. Am. J. Geriatr. Psychiatry 28, 835–838. doi: 10.1016/j.jagp.2020.04.025, PMID: 32430111 PMC7196899

[ref21] Jeong M. Kim K. Ma F. DiPietro R. (2022). Key factors driving customers’ restaurant dining behavior during the COVID-19 pandemic. IJCHM 34, 836–858. doi: 10.1108/IJCHM-07-2021-0831

[ref22] Ju J. Oh E. Kim J. (2014). A research on the improvement of dining space design at elderly welfare facility. J. Korea Inst. Healthcare Archit. 20, 39–48. doi: 10.15682/jkiha.2014.20.4.39

[ref23] Keller H. Carrier N. Duizer L. Lengyel C. Slaughter S. Steele C. (2014). Making the most of mealtimes (M3): grounding mealtime interventions with a conceptual model. J. Am. Med. Dir. Assoc. 15, 158–161. doi: 10.1016/j.jamda.2013.12.001, PMID: 24513225 PMC4316206

[ref24] Kenkmann A. Hooper L. (2012). The restaurant within the home: experiences of a restaurant-style dining provision in residential homes for older people. Qual. Ageing 13, 98–110. doi: 10.1108/14717791211231184

[ref25] Kim J. Kim Y. Ha J. (2021). Changes in daily life during the COVID-19 pandemic among south Korean older adults with chronic diseases: a qualitative study. Int. J. Environ. Res. Public Health 18:6781. doi: 10.3390/ijerph18136781, PMID: 34202534 PMC8297182

[ref26] Liu Y. Jang S. S. (2009). The effects of dining atmospherics: an extended Mehrabian–Russell model. Int. J. Hosp. Manag. 28, 494–503. doi: 10.1016/j.ijhm.2009.01.002

[ref27] McDaniel J. H. Hunt A. Hackes B. Pope J. F. (2001). Impact of dining room environment on nutritional intake of Alzheimer’s residents: a case study. Am. J. Alzheimer’s Dis. Other Demen. 16, 297–302. doi: 10.1177/153331750101600508 11603166 PMC10833876

[ref29] Meng Q. Sun Y. Kang J. (2017). Effect of temporary open-air markets on the sound environment and acoustic perception based on the crowd density characteristics. Sci. Total Environ. 601–602, 1488–1495. doi: 10.1016/j.scitotenv.2017.06.017, PMID: 28605866

[ref30] Meng Q. Zhang S. Kang J. (2017). Effects of typical dining styles on conversation behaviours and acoustic perception in restaurants in China 121, 148–157.

[ref31] Mokros H. B. Deetz S. (2013). “What counts as real? a constitutive view of communication and the disenfranchised in the context of health” in Communication and Disenfranchisement. ed. Ray E. B. (New York: Routledge), 51–66.

[ref32] Morley J. E. Silver A. J. (1995). Nutritional issues in nursing home care. Ann. Intern. Med. 123, 850–859. doi: 10.7326/0003-4819-123-11-199512010-00008, PMID: 7486469

[ref33] Morrow-Howell N. Galucia N. Swinford E. (2020). Recovering from the COVID-19 pandemic: a focus on older adults. J. Aging Soc. Policy 32, 526–535. doi: 10.1080/08959420.2020.1759758, PMID: 32336225

[ref34] Mu J. Kang J. (2022). Indoor environmental quality of residential elderly care facilities in Northeast China. Front. Public Health 10:860976. doi: 10.3389/fpubh.2022.860976, PMID: 35602153 PMC9116475

[ref35] Mullol J. Alobid I. Marino-Sanchez F. Quinto L. de Haro J. Bernal-Sprekelsen M.. (2012). Furthering the understanding of olfaction, prevalence of loss of smell and risk factors: a population-based survey (OLFACAT study). BMJ Open 2:e001256. doi: 10.1136/bmjopen-2012-001256, PMID: 23135536 PMC3533119

[ref36] Pauly L. Stehle P. Volkert D. (2007). Nutritional situation of elderly nursing home residents. Z. Gerontol. Geriatr. 40, 3–12. doi: 10.1007/s00391-007-0430-x, PMID: 17318726

[ref37] Pizzola L. Martos Z. Pfisterer K. de Groot L. Keller H. (2013). Construct validation and test–retest reliability of a mealtime satisfaction questionnaire for retirement home residents. J. Nutr. Gerontol. 32, 343–359. doi: 10.1080/21551197.2013.840257, PMID: 24224941

[ref38] Realyvásquez-Vargas A. Maldonado-Macías A. A. Arredondo-Soto K. C. Baez-Lopez Y. Carrillo-Gutiérrez T. Hernández-Escobedo G. (2020). The impact of environmental factors on academic performance of university students taking online classes during the COVID-19 pandemic in Mexico. Sustainability 12:9194. doi: 10.3390/su12219194

[ref39] Rockwood K. Song X. MacKnight C. Bergman H. Hogan D. B. McDowell I.. (2005). A global clinical measure of fitness and frailty in elderly people. J. Assoc. Med. Can. 173, 489–495. doi: 10.1503/cmaj.050051, PMID: 16129869 PMC1188185

[ref40] Salamone F. Barozzi B. Bellazzi A. Belussi L. Danza L. Devitofrancesco A.. (2021). Working from home in Italy during COVID-19 lockdown: a survey to assess the indoor environmental quality and productivity. Buildings 11:660. doi: 10.3390/buildings11120660

[ref41] Sidenvall B. (1999). Meal procedures in institutions for elderly people: a theoretical interpretation. J. Adv. Nurs. 30, 319–328. doi: 10.1046/j.1365-2648.1999.01082.x, PMID: 10457233

[ref42] Stroebele N. De Castro J. M. (2004). Effect of ambience on food intake and food choice. Nutrition 20, 821–838. doi: 10.1016/j.nut.2004.05.012, PMID: 15325695

[ref43] Stuck A. E. Aronow H. U. Steiner A. Alessi C. A. Bula C. J. Gold M. N.. (1995). A trial of annual in-home comprehensive geriatric assessments for elderly people living in the community. N. Engl. J. Med. 333, 1184–1189. doi: 10.1056/NEJM199511023331805, PMID: 7565974

[ref44] Sun C. Zhai Z. (2020). The efficacy of social distance and ventilation effectiveness in preventing COVID-19 transmission. Sustain. Cities Soc. 62:102390. doi: 10.1016/j.scs.2020.102390, PMID: 32834937 PMC7357531

[ref45] Sylvie A. K. Jiang Q. Cohen N. (2013). Identification of environmental supports for healthy eating in older adults. J. Nutr. Gerontol. Geriatr. 32, 161–174. doi: 10.1080/21551197.2013.779621, PMID: 23663214

[ref46] Trichopoulou A. Kouris-Blazos A. Wahlqvist M. L. Gnardellis C. Lagiou P. Polychronopoulos E.. (1995). Diet and overall survival in elderly people. BMJ Open 311, 1457–1460. 10.1136/bmj.311.7018.1457 PMC2543726 8520331

[ref47] Triyason T Tassanaviboon A Kanthamanon P. (2020). Hybrid classroom: designing for the new normal after COVID-19 pandemic. In Proceedings of the 11th International Conference on Advances in Information Technology 1–8.

[ref48] Visser M. Schaap L. A. Wijnhoven H. A. H. (2020). Self-reported impact of the COVID-19 pandemic on nutrition and physical activity behaviour in Dutch older adults living independently. Nutrients 12:3708. doi: 10.3390/nu12123708, PMID: 33266217 PMC7760336

[ref49] Wang D. Yao J. Martin B. A. (2021). The effects of crowdedness and safety measures on restaurant patronage choices and perceptions in the COVID-19 pandemic. Int. J. Hosp. Manag. 95:102910. doi: 10.1016/j.ijhm.2021.102910, PMID: 36540677 PMC9756833

[ref50] Wu C.-C. Lin W.-L. (2022). Development of evaluation indicators for senior-friendly restaurants. Br. Food J. doi: 10.1108/BFJ-11-2021-1264

[ref51] Xie K. Liang B. Dulebenets M. A. Mei Y. (2020). The impact of risk perception on social distancing during the COVID-19 pandemic in China. Int. J. Environ. Res. Public Health 17:6256. doi: 10.3390/ijerph17176256, PMID: 32867381 PMC7503995

