# Supplementary figures and images for: Dining comfort in elderly care facility dining rooms and influencing factors before and after the outbreak of the COVID-19 pandemic
Source: Front Psychol. 2023 Mar 3;14:1106741. doi: 10.3389/fpsyg.2023.1106741 (PMC10022735; doi:10.3389/fpsyg.2023.1106741)

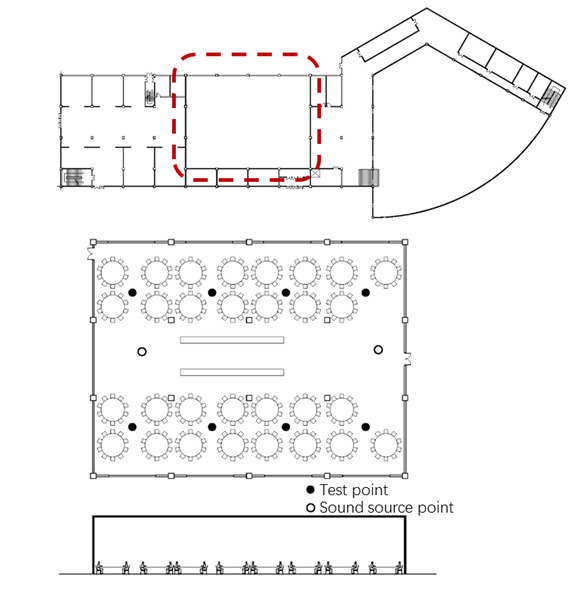

Supplement: Supplementary file 1 [file Image_1.tif]

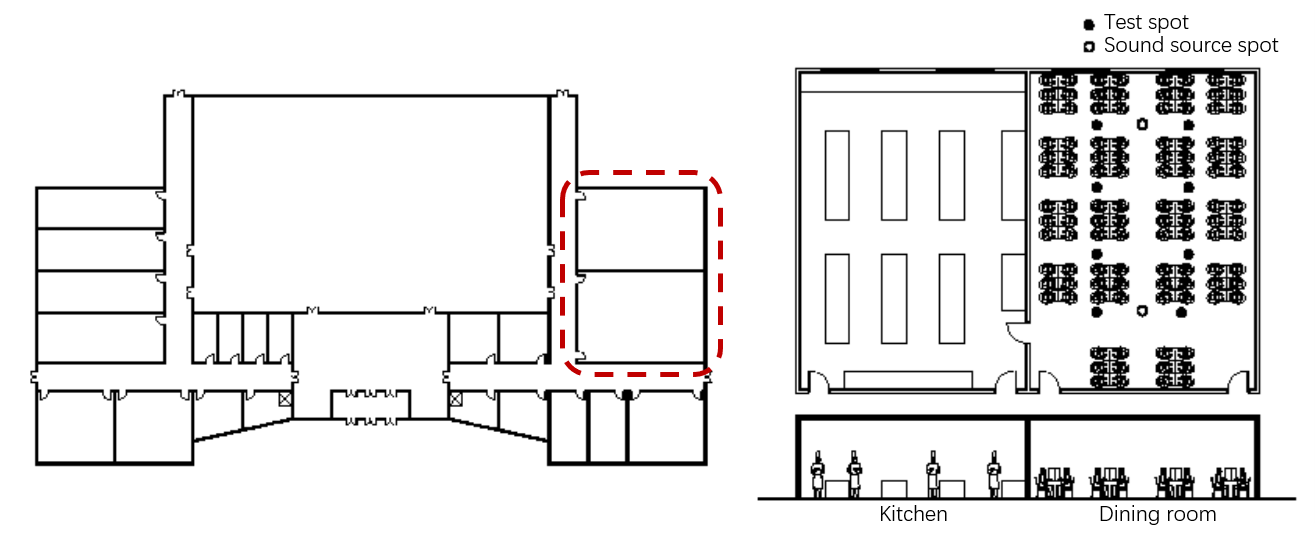

Supplement: Supplementary file 2 [file Image_2.tif]

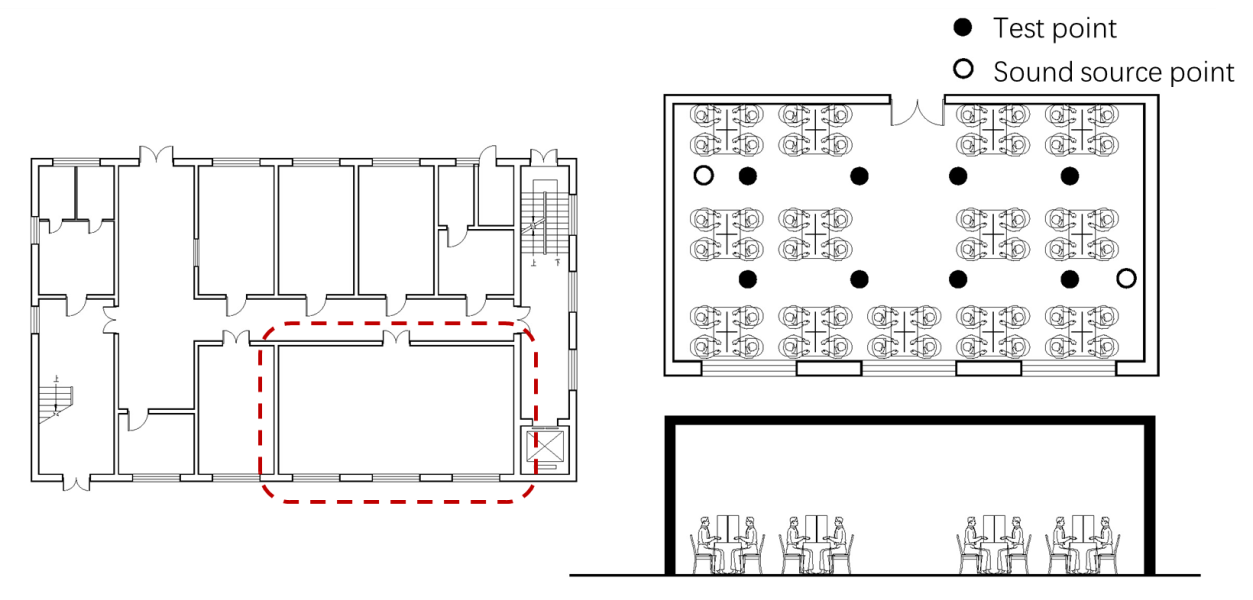

Supplement: Supplementary file 3 [file Image_3.tif]

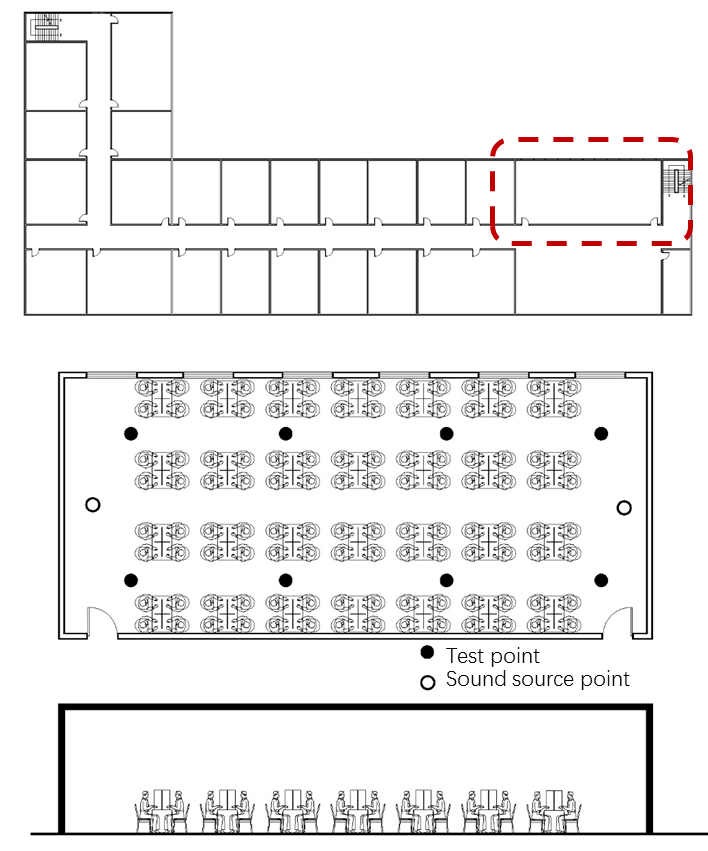

Supplement: Supplementary file 4 [file Image_4.tif]
